# Correlates of Integrated Human Papillomavirus Vaccination and Cervical Cancer Screening Protection in U.S. Low-Income Women

**DOI:** 10.3390/vaccines14030251

**Published:** 2026-03-09

**Authors:** Erika B. Biederman, Victoria L. Champion, Katharine J. Head, Teresa M. Imburgia, Gregory D. Zimet

**Affiliations:** 1 Department of Community Health Systems, Indiana University School of Nursing, Indianapolis, IN 46202, USA; vchampio@iu.edu; 2 Department of Communication Studies, Indiana University-Indianapolis, Indianapolis, IN 46202, USA; headkj@iu.edu; 3 Department of Epidemiology, Richard M. Fairbanks School of Public Health, Indianapolis, IN 46202, USA; tcumming@iu.edu; 4 Indiana University School of Medicine, 1625 Sturbridge Road, Indianapolis, IN 46260, USA; gzimet@iu.edu

**Keywords:** human papillomavirus vaccination, cervical cancer screening, health disparities

## Abstract

Background/Objectives: In the United States, adult human papillomavirus (HPV) vaccination coverage remains low at 20–50%, depending on age, and cervical cancer (CC) screening rates range from 68 to 76%. Few studies have evaluated characteristics of women who are both HPV vaccinated and up to date (UTD) with screening as an integrated outcome. The purpose of the present study was to classify women into four prevention categories and examine factors associated with being double protected compared to unprotected. Methods: Data were gathered via an online survey from a sample of low-income women (household income < USD 50,000) provided by a research survey company (n = 719). Women were classified into four categories: vaccinated only, screened only, both vaccinated and screened (double protected), or neither (unprotected). Sociodemographic characteristics, healthcare access, and Health Belief Model constructs were assessed. Multivariable logistic regression compared women who were double protected with those unprotected (n = 274). Results: Most women were UTD with screening only (57.8%), while 15.5% were double protected and 22.6% were unprotected. Younger age (Odds Ratio [OR = 0.93; 95% Confidence Interval [CI]: 0.89, 0.98), having ≥1 medical visit in the past year (OR = 4.16; 95% CI: 1.74, 9.95), higher perceived CC risk (OR = 3.65; 95% CI: 1.41, 9.43), greater perceived benefits of CC screening (OR = 1.96; 95% CI: 1.45, 2.66), and higher HPV knowledge (OR = 1.09; 95% CI: 1.01, 1.17) were associated with higher odds of being double protected. Conclusions: A substantial proportion of low-income women lack comprehensive CC prevention. Integrated, bundled prevention strategies that simultaneously promote HPV vaccination and screening may be important to reduce CC disparities.

## 1. Introduction

Cervical cancer (CC) results in an estimated 4320 deaths and nearly 13,360 new invasive cases annually in the United States (U.S.) [1]. Women residing in high-poverty communities experience almost 1.5–2- times higher incidence and nearly double the mortality in low-poverty counties [2,3]. While these estimates reflect area-level poverty, they provide relevant context for the current study, which focuses on women with low-income (annual income below USD 50,000) [4,5]. Prevention of CC relies on both primary prevention through human papillomavirus (HPV) vaccination and secondary prevention through CC screening. However, persistent disparities in vaccination and screening coverage continue to limit progress toward CC elimination. Although approximately 78% of adolescent females have received at least one dose of the HPV vaccine [6], vaccination uptake among adults remains substantially lower, with only about 58% of women aged 19–26 [7] and 16% of women aged 27–45 having received at least one dose [4]. Additionally, CC screening rates vary by population; approximately 76% of non-Hispanic White women are up to date (UTD) with screening, compared with roughly 68% of Hispanic and low-income women [8]. Furthermore, HPV testing use remains modest, with an estimated uptake of around 52% [9].

To achieve global CC elimination targets, the World Health Organization (WHO) recommends that 90% of girls be fully vaccinated against HPV by age 14 and that 70% of women undergo HPV-based screening at least twice in their lifetime [10]. In the U.S., many women fall into a “transition cohort,” those who were too old to benefit from routine adolescent HPV vaccination and who have not yet received HPV-based CC screening, leaving them insufficiently protected against CC. In the U.S., the U.S. Preventive Service Task Force (USPSTF) guidelines continue to still recommend Papanicolaou (Pap) testing every three years or co-testing (Pap and HPV testing) every five years [11]. Women who are underserved, especially low-income, likely benefit the most from being both HPV vaccinated and UTD with screening as a lower proportion of them are likely to report ever having a HPV test [12] and have greater barriers to follow-up for an abnormal Pap smear [13].

Most studies examining factors associated with HPV vaccination and CC screening have evaluated these behaviors separately and conceptualized them as distinct outcomes [14,15]. However, these preventive behaviors share overlapping determinants and may function synergistically [16,17]. For example, prior research has demonstrated that receipt of the HPV vaccine is associated with increased uptake of CC screening [16,18]. Additionally, several factors, including healthcare access, age, race/ethnicity, perceived risk of CC, perceived benefits, and HPV knowledge, have been consistently associated with HPV vaccination and being UTD with CC screening independently [19,20,21,22]. Women who are low-income experience structural and personal barriers that make them particularly vulnerable to CC and follow-up treatment so that prevention of disease is critical in this population [13]. HPV vaccination is associated with a reduced risk of invasive CC [23] while women who are not UTD with screening bear a disproportionate share of CC deaths [24]. Consequently, women who are neither vaccinated nor UTD with screening represent the population at highest risk for preventable CC morbidity and mortality [25].

Comparing women who are “double protected” (defined as having received at least one dose of the HPV vaccine [26] and being UTD with CC screening [11]) with those who are “unprotected” (women who have not received the HPV vaccine and are not UTD with screening) provides insight into who should be prioritized for intervention and which modifiable factors may be most responsive to behavioral change. Double protected and unprotected are terms used in the current study as analytic group labels. Although HPV vaccination and CC screening share similar determinants, few studies have examined interventions designed to simultaneously target both behaviors. However, previous studies that have examined determinants of being UTD with multiple screening behaviors such as breast and CC screening show that preventive behaviors may cluster together [27,28] and that bundled preventive behavior interventions may be particularly effective. Specifically, a behavioral intervention that integrated screening for multiple cancers, including breast, cervical, and colorectal cancer, resulted in women having two- to six-fold greater odds (depending on intervention arm) of being UTD with screening for all cancers compared with usual care [29]. These findings support the premise that bundling preventive behaviors within a single intervention may yield greater impact than addressing each behavior independently.

The purpose of the current study was twofold. First, low-income women were classified into four CC prevention categories: (1) receipt of at least one dose of HPV vaccine only (not UTD with CC screening), (2) UTD with CC screening (no receipt of HPV vaccine, (3) receipt of at least one dose of the HPV vaccine and UTD with CC screening (double protected), and (4) receipt of neither HPV vaccine nor UTD with CC screening (unprotected). Second, factors associated with being double protected were examined by comparing women who had received at least one dose of the HPV vaccine and UTD with CC screening to those who had received neither preventive service. Understanding the characteristics of women who are fully protected compared with those who are unprotected against CC may help clinicians and public health researchers more effectively identify priority populations for targeted prevention efforts and aid with the development of tailored integrated HPV vaccine and CC screening interventions. This approach captures the continuum of CC prevention and aligns with a framework that emphasizes integrated primary and secondary prevention.

## 2. Materials and Methods

### 2.1. Sample and Study Design

This study was reviewed and approved as exempt research by the university Institutional Review Board (IRB). Cross-sectional data were collected in August 2020 using an online survey administered by a survey research company (Dynata). The parent dataset included women aged 21–65 years. For the current analysis, the analytic sample was restricted to women aged 21–49 years. This age range was selected because it aligns with USPSTF recommendations for CC screening [11] and reflects the age range in which women were most likely to have received the HPV vaccine, given that the U.S. Food and Drug Administration (FDA) expanded vaccine eligibility to include adults aged 27–45 years in 2018 [30].

Eligibility criteria for the analytic sample included women who reported an annual household income of less than USD 50,000, were able to read and write in English, and had not undergone a hysterectomy. Annual household income <USD 50,000 was used to classify participants as lower income, consistent with prior research using this threshold as a proxy for financial constraint when family-size data are unavailable [4,5]. In the U.S., USD 50,000 annual income approximates ≤200–300% of the federal poverty guideline for many household sizes, although the purchasing power of a given income varies by household size and cost of living [31]. To ensure robust analysis of populations disproportionately affected by CC disparities [32], we used an oversampling strategy. Rather than strictly mirroring the U.S. Census distribution, we intentionally oversampled women identifying as Black/African American and Hispanic/Latina. E-mail invitations were sent by Dynata to the target demographics. After clicking a link to an online survey through Qualtrics (Qualtrics, Provo, Utah), women completed eligibility criteria. If eligible, participants read a brief description of the study, and if they chose to participate, were directed to the survey.

### 2.2. Measures

#### 2.2.1. Sociodemographics and Healthcare Access

Sociodemographics were collected using standard self-reported measures. Characteristics included age (continuous, in years), race, and ethnicity. Race and ethnicity were self-reported and collected in accordance with National Institutes of Health (NIH) standards. Since a small number of participants were not Hispanic, non-Hispanic Black, or non-Hispanic White, other races were collapsed into the non-Hispanic White category. Healthcare access was assessed using two self-reported measures that collapsed into dichotomous categories. Current health insurance status was assessed as insured vs. uninsured and any medical visit in the past year, excluding dental and eye visits (yes/no).

#### 2.2.2. Health Belief Model (HBM) Constructs

Perceived risk was measured with a single validated item: “Compared to other women your same age, would you say your chance of getting cervical cancer in the next 10 years is higher, about the same, or lower.” Responses of “about the same” and “lower” were collapsed into a single category and compared to “higher.” Similar single item perceived risk measures have been validated in previous research [33]. Perceived benefits (operationalized as perceived importance) was assessed with one item: “How important is it for you to get checked for cervical cancer?” Responses were measured on a 5-point Likert scale ranging from “not at all important” to “very important.” Similar single-item measures of perceived importance have been used as indicators of perceived benefits in prior cancer screening research [34,35]. HPV knowledge was assessed using 15 true/false/don’t know items adapted from the validated Waller HPV knowledge scale [36]. These items were scored and summed with a higher score indicating higher HPV knowledge. Internal consistency was strong in the current sample (Cronbach’s α = 0.85).

#### 2.2.3. CC Prevention History

HPV vaccination history was assessed with a single question if they had received at least one dose of the HPV vaccine (yes/no). HPV vaccination was operationalized as receipt of at least one dose, rather than completion of the full vaccination series. HPV vaccination as receipt of at least one dose is consistent with prior research examining adult HPV vaccination patterns, including national studies using National Health Information Survey and Behavioral Risk Factor Surveillance System data that assess HPV vaccination as receipt of at least one dose [37,38]. Evidence suggests that a single dose of the HPV vaccine may give considerable protection against HPV infection with one study reporting 80.2% vaccine efficacy against prevalent HPV16 or 18 infection [39]. Self-reported UTD with CC screening was assessed with branching logic that allowed participants to identify their last CC screening test (Pap alone vs. co-testing vs. HPV testing) and timing of the test with choices ranging from never to less than 3 years ago. Choices were collapsed into being UTD, which was defined according to 2018 USPSTF guidelines with Papanicolaou (Pap) alone as less than 3 years ago and HPV/co-testing as less than 5 years ago [11].

#### 2.2.4. Double Protected vs. Unprotected Outcome

A combined CC prevention outcome was created with HPV vaccine receipt history (yes/no) and UTD CC screening status (yes/no). Women were classified as “double protected” if they reported receipt of at least one dose of the HPV vaccine and being UTD with CC screening. Women were classified as “unprotected” if they reported being neither receipt of at least one dose of the HPV vaccine or being UTD with CC. These two groups served as the primary analytic outcome for the current study.

### 2.3. Analyses

Four mutually exclusive CC prevention categories were calculated: vaccinated only, screened only, both vaccinated and screened (double protected), and neither vaccinated nor screened (unprotected). Descriptive statistics were calculated to summarize participant characteristics overall, and differences between being double protected vs. unprotected were assessed using chi-square tests for categorical variables and independent samples *t* tests for continuous variables, as appropriate.

For multivariable analyses, the primary outcome was defined as being double protected vs. unprotected. An extreme groups approach was taken that increases power to detect separate characteristics [40]. Logistic regression analyses were conducted to evaluate associations between sociodemographic factors, healthcare access, and HBM constructs with the odds of being double protected. Variables of *p* < 0.10 in bivariable analyses were entered into the multivariable logistic regression model. Odds ratios (ORs) and 95% confidence intervals (CIs) were estimated. Model diagnostics were assessed to evaluate multicollinearity and overall model fit. Statistical significance was defined as *p* < 0.05, and all analyses were performed using IBM Statistical Package for Social Sciences (SPSS) Version 31.

## 3. Results

Among women aged 21–49 (n = 719), 19.7% reported receipt of at least one dose of the HPV vaccine, and 73.3% were UTD with CC screening. The percentage of women in the four categories of vaccinated and screened are described in Table 1. The largest proportion of women were UTD with CC screening only (n = 415, 57.8%). Smaller proportions were double protected by being both UTD with screening and vaccinated (n = 111, 15.5%), unprotected by being neither screened nor vaccinated (n = 162, 22.6%) or had received HPV vaccination only (n = 30, 4.2%).

The sample sizes within each of the four prevention categories were small, particularly the HPV-vaccinated-only group (n = 30), which was insufficient for stable multivariable modeling given recommended participants per variable thresholds of 10 per variable [41]. Subsequent analyses focused on a subset of women (n = 274) representing the extremes of prevention status: those who were double protected (vaccinated and UTD with CC screening) and those who were minimally protected (neither screened nor vaccinated).

Descriptive and bivariate results are presented in Table 2. Within this subset, the mean age was 38.05 years (SD = 6.53; range = 21.49). Most participants were insured (n = 222, 81.9%) and reported at least one medical visit in the past year (n = 208, 75.9%). The sample was racially and ethnically diverse: 29.0% (n = 78) identified as Hispanic, 35.0% (n = 97) as non-Hispanic Black, and 36.0% (n = 99) as non-Hispanic White/Other. Most participants (86.9%, n = 232) perceived themselves to be at the same or lower risk for CC compared to other women their age. The mean perceived benefits score was 4.01 (SD = 1.08; range = 1.5), and the mean HPV knowledge score was 8.03 (SD = 4.16; range = 0.15).

Bivariate analyses comparing women who were double protected with those who were unprotected showed significant differences across multiple characteristics. Women who were double protected were younger on average (mean [M] = 36.23 years, SD = 5.63) compared to unprotected women (M = 39.30 years, SD = 6.82; *p* < 0.001). A smaller percentage, 16% (n = 8), of those double protected were uninsured compared to 84% (n = 41) of those unprotected (*p* < 0.001). Similarly, fewer double-protected women reported no medical visit in the past year (12.0%, n = 8) compared to unprotected women (88.0%, n = 58; *p* < 0.001). Racial and ethnic distributions differed between groups. Among women who were double protected, 47.0% (n = 37) identified as Hispanic compared to 52.6% (n = 41) of unprotected women. In the double-protected group, 41.0% (n = 40) identified as non-Hispanic Black and 34.0% (n = 34) as non-Hispanic White/Other, compared to 58.8% (n = 57) non-Hispanic Black and 65.7% (n = 65) non-Hispanic White/Other among unprotected women. A significantly greater proportion of double-protected women reported higher perceived risk of CC compared to unprotected women (68.6% [n = 24] vs. 36.3% [n = 11], *p* < 0.001). Mean perceived benefits scores were also higher among double-protected women (M = 4.49, SD = 0.83) than among unprotected women (M = 3.69, SD = 1.12; *p* < 0.001). HPV knowledge scores were significantly higher in the double-protected group (M = 9.07, SD = 3.80) compared to the unprotected group (M = 7.33, SD = 4.25; *p* < 0.001).

Multivariable logistic regression analyses (Table 3) indicated that younger age (odds ratio [OR] = 0.93; 95% confidence interval [CI]: 0.89, 0.98; *p* = 0.003), ≥1 medical visit in the past year (OR = 4.16; 95% CI: 1.74, 9.95; *p* = 0.001), higher perceived CC risk (OR = 3.65; 95% CI: 1.41, 9.43; *p* = 0.008), greater perceived benefits of CC screening (OR = 1.96; 95% CI: 1.45, 2.66; *p* < 0.001), and higher HPV knowledge scores (OR = 1.09; 95% CI: 1.01, 1.17; *p* = 0.032) were associated with higher odds of being double protected. Being insured was not significantly associated with being double protected in the adjusted model. Multicollinearity diagnostics showed no collinearity between co-variates (all variance inflation factors ≤ 1.21).

## 4. Discussion

The current study extends prior work that has examined HPV vaccination as a predictor of CC screening (or vice versa) by evaluating factors associated with being double protected, defined as receipt of at least one dose of the HPV vaccine and being up to date with CC screening [16,18]. Rather than applying vaccination and screening as individual outcomes, this study sought to evaluate factors related to double protection among U.S. low-income women aged 21–49 years. Overall, almost 20% of the women in our sample had received the HPV vaccine, which is in line with a recent study that found almost 20% of women aged 27–45 years had been vaccinated in national U.S. Behavioral Risk Factor Surveillance System (BRFSS) data [42]. Our overall screening rate was slightly higher (73%) compared to the 67.7% screening coverage reported in that same previous study, potentially reflecting racial/ethnic differences in the population in the current study, slightly different age cohort sampled (21–49 years in the current study vs. 27–45 years in previous studies), and that HPV testing/co-testing was included as a screening modality in the current study [42]. These findings demonstrate persistent gaps in adult HPV vaccination uptake while highlighting the importance of examining vaccination and screening jointly to identify women who remain incompletely protected across the CC prevention continuum.

The first aim of our study evaluated the percentage of women in four categories of prevention: (1) receipt of at least one dose of HPV vaccine only (not UTD with CC screening), (2) UTD with CC screening only (no receipt of HPV vaccine), (3) UTD with CC screening and at least one dose of the HPV vaccine (double protected), and (4) receipt of neither HPV vaccine nor UTD with CC screening (unprotected). Our finding that most participants were UTD with screening only and the fewest had received an HPV vaccine only is in line with higher population coverage of CC screening than HPV vaccination among adults and that HPV vaccination and CC screening cluster [18,42]. Vaccinated women are significantly more likely to be UTD with screening, whereas unscreened, unvaccinated women represent a concentrated high-risk group [18,42]. Our second aim was to evaluate the characteristics associated with being double protected vs. unprotected in bivariate and multivariable analysis. In contrast to prior BRFSS work limited to sociodemographic predictors [12,30], the present analyses incorporated theoretically relevant HBM constructs that are potentially modifiable through intervention [18,42].

The sociodemographic characteristics evaluated in the current study included age and race/ethnicity. Age was found to be significant in both bivariate and multivariable analyses. In previous studies, younger age is often associated with HPV vaccination [14,43] while older age is associated with CC screening [44,45] reflecting differences in guidelines and cohort eligibility. Many of the older participants in this sample may have missed the window for HPV vaccination, were unaware of expanded eligibility, and/or faced financial barriers to vaccination with variable insurance coverage for ages 27–45 [46]. Race/ethnicity was not found to be related to being double protected in this study; however, one might expect non-Hispanic White women to have greater odds of being double protected based on national U.S. data survey that show higher CC screening coverage [47] and adult HPV vaccination uptake [37] relative to other racial/ethnic groups. Age distribution and/or differences in healthcare access may modify the relationship between race/ethnicity and double protection in this sample, and this relationship should be explored in a larger sample size.

Healthcare access variables evaluated in this study included insurance status and any medical visits in the past year. Insurance status was found to be significantly associated with double protection in both bivariate and multivariable analysis. This result aligns with a previous study using national U.S. data that found uninsured women had lower odds of both HPV vaccination (OR = 0.48) and being UTD with screening (OR = 0.41) [18]. Because the HPV vaccine can cost up to USD 350 per dose without insurance, out-of-pocket cost likely represents a significant barrier to achieving full protection [48]. Greater healthcare utilization would be expected to increase the likelihood of being double protected because more frequent contact with the healthcare system is associated with both higher HPV vaccination uptake and greater CC screening participation [44,49]. Additionally, greater healthcare utilization allows for more opportunities for provider recommendation of the HPV vaccine [14] and CC screening [50] as well as barriers counseling, which were not measured in the current study but should be considered in future research. Although insurance status was not significant in the multivariable model, recent healthcare utilization remained associated with double protection. Multicollinearity was not found between insurance coverage and healthcare utilization. These findings suggest that insurance and utilization represent distinct aspects of healthcare access [51]. Healthcare utilization could be a more proximal determinant of HPV vaccination and CC screening, potentially mediating the relationship between insurance coverage and prevention behaviors, which should be evaluated in a future study with a larger sample size.

All the HBM constructs in the present study, including perceived risk of CC, perceived benefits of screening, and HPV knowledge, were associated with being double protected. Although multi-item scales are often preferred, single-item measures have been shown to perform adequately for concepts such as risk and importance of screening. Single-item scales can reduce participant burden and improve survey completion rates among underserved populations [52]. A review found that perceived risk of developing CC has been significantly related to HPV vaccination uptake among adults in multiple studies [53]. Similarly, a three-item scale of perceived risk of CC was positively associated with CC screening uptake in low-income women in North Carolina, suggesting that perceived risk may be a particularly salient factor for screening in underserved populations [22]. In that same study, a three-item “positive perceptions of screening” scale included an item like the perceived benefits measure used in the current study and was significantly associated with a screening attempt (adjusted OR = 2.51). Perceived benefits related to vaccination have also been associated with HPV vaccine uptake among college-aged women [54]. Although the current study assessed perceived benefits with a single item specific to CC screening (rather than both vaccination and screening), our findings contribute to the emerging literature indicating that CC prevention is important to women who achieve double protection. While the specific HPV knowledge scale used in the present study has not been widely tested among U.S. adult populations to our knowledge, HPV knowledge has been consistently associated with HPV vaccine uptake [46]. Further, while HPV knowledge has infrequently been correlated with Pap testing, it has been associated with HPV testing uptake [21].

Collectively, these findings suggest that healthcare access, perceived risk, perceived benefits, and HPV knowledge could be actionable in a bundled intervention to increase HPV vaccination and CC screening among low-income women. Reminders could be used by clinicians to encourage healthcare visits [55]. Future research should investigate the types of reminders that low-income women prefer, e.g., phone call, text message, or mailed letter, and which are preferred and most effective for this population [56]. In addition, newer screening modalities such as self-collection for HPV testing could increase access to screening [57], as healthcare visits were positively associated with double protection. Tailored messaging based on the HBM constructs may also strengthen intervention impact [29]. For example, women indicating low perceived risk could receive information emphasizing CC and HPV risk factors, e.g., HPV infection, smoking, sexual history, low socioeconomic status [58]. Women expressing low perceived benefits to CC screening could receive messages emphasizing the importance and value of early detection and personal health. Women demonstrating limited HPV knowledge could receive brief education regarding HPV transmission and prevention. Because the perceived risk and benefit measures used in this study were efficient single-item instruments, clinicians could feasibly use similar items in practice to rapidly identify women at elevated risk for being unprotected.

## 5. Limitations

The current study has several limitations. First, the sample sizes within each of the four prevention categories were small, particularly the HPV-vaccinated-only group, preventing multivariable analyses across all categories. By only analyzing the extremes of prevention, generalization is limited to women with limited protection (i.e., vaccinated only or UTD with screening only). Future research using larger samples should examine correlates of each specific category (HPV vaccinated only, UTD with screening only, double protected, and unprotected). Determinants of partial protection may differ from the associations found here with double protection. For example, women who are UTD with screening but unvaccinated may be influenced primarily by structural barriers to vaccination (e.g., insurance coverage, cost, age eligibility), whereas women who are vaccinated but not UTD with screening may have psychosocial barriers related to screening like pain or embarrassment.

Future studies should also evaluate determinants of HPV testing and HPV vaccination, which together represent the strongest levels of prevention recommended by the WHO [10]. Additionally, the current study did not assess if women had received both doses of the HPV vaccine, which is the strongest level of vaccination protection [10]. Nonetheless, an important strength of this study was the focus on factors associated with being double protected versus unprotected, which could inform future bundled HPV vaccination and CC screening interventions for adult women. Future research could also consider examining factors related to a mother being UTD with screening and having her children vaccinated against HPV [52] for a bundled mother/child intervention [53]. Because the strongest variable in our model was healthcare utilization, prioritizing settings with regular contact (e.g., primary care, OB/GYN) may be the best way to bundle a mother-child intervention when a mother is already present for her own care.

Second, the study sample consisted of low-income women and intentionally oversampled Black and Hispanic participants. Although a national survey company was used, the resulting sample is not nationally representative, and findings may not generalize to broader populations. For example, non-Hispanic White and higher-SES populations may exhibit different vaccination patterns or correlates of uptake than those observed here. However, the low-income, racially/ethnically diverse population represented in this study has historically been underrepresented in CC prevention research and is at elevated risk for being neither vaccinated nor screened [59,60]. A strength of this sampling strategy was the ability to assess HBM constructs that are not routinely captured in national surveillance data but could be actionable intervention targets.

Third, HBM constructs (i.e., perceived risk and benefits) were measured with a single item, which may limit construct validity and may not fully capture the multidimensionality of these constructs [52]. As a result, ORs should be interpreted cautiously. Despite these limitations, these single-item scales demonstrated expected associations with double protection and aligned with prior study findings, supporting criterion validity [22,53,54]. However, future research should consider multiple faceted, multiple item scales rather than single items. Additionally, the study did not include several HBM and Theory of Planned Behavior variables known to influence HPV vaccination and CC screening behavior, such as provider recommendation, perceived barriers, self-efficacy, or behavioral intention [61,62,63]. Future research should incorporate such determinants to provide a more comprehensive framework of determinants of double protection. Additionally, measures of perceived benefits should include both CC screening and vaccination. Fourth, the cross-sectional design limits conclusions regarding temporality or causality. Associations observed between healthcare utilization, HBM constructs, and double protection cannot determine whether these factors came before or resulted from HPV vaccination or CC screening behaviors. Finally, outcomes were based on participant self-report, which may have resulted in incorrect or overestimates of vaccination or screening rates [64]. However, the estimates were consistent with prior studies using national data sources.

## 6. Conclusions

This study of low-income U.S. women showed that a small proportion were double protected against CC through being vaccinated against HPV and UTD with CC screening, while a larger proportion remained unprotected, meaning they had never received an HPV vaccination nor were UTD with screening. Women who were double protected were more likely to be younger, have recent healthcare utilization, perceive higher CC risk, perceive higher benefits of CC screening, and have greater HPV knowledge. These factors are potentially modifiable through intervention. However, because sample sizes within prevention categories were small and analyses focused on the extremes (double protected vs unprotected), findings should be interpreted cautiously and may not generalize to women with partial protection (HPV vaccinated only or CC screening only). Findings support a bundled CC prevention approach that integrates HPV vaccination and screening using theory-based messaging to address health beliefs. Prioritizing women in interventions who are unprotected with integrated primary and secondary prevention strategies may be important to reduce CC disparities and advance CC elimination efforts.

## Figures and Tables

**Table 1:** Cervical cancer prevention status among women aged 21–49 (n = 719).

Prevention Category	N (%)
Receipt of HPV vaccine only (not UTD with screening)	30 (4.2)
UTD with CC screening only (no receipt of HPV vaccine)	415 (57.8)
Double protected (UTD with CC screening and receipt of HPV vaccine)	111 (15.5)
Unprotected (neither UTD with screening nor receipt of HPV vaccine)	163 (22.7)

Notes: Receipt of HPV vaccine reflects ≥1 dose. UTD with CC screening was defined as Pap testing within the past 3 years or HPV testing within the past 5 years. Abbreviations: CC = cervical cancer; HPV = human papillomavirus; UTD = up to date.

**Table 2:** Descriptive and bivariate characteristics associated with being double protected vs. unprotected.

Characteristic	Total (n = 274)	Unprotected (n = 163)	Double Protected (n = 111)	*p*-Value
Age M (SD)	38.05 (6.53)	39.30 (6.82)	36.23 (5.63)	<0.001
Insured n (%)				<0.001
No	49 (18.10)	41 (83.7)	8 (16.3)	
Yes	222 (81.90)	120 (54.1)	102 (45.9)	
≥1 medical visit in the past year n (%)				<0.001
No	66 (24.1)	58 (87.9)	8 (12.1)	
Yes	208 (75.9)	105 (50.5)	103 (49.5)	
Race/Ethnicity n (%)				0.208
Hispanic (any race)	78 (28.5)	41 (52.6)	37 (47.4)	
Non-Hispanic Black	97 (35.4)	57 (58.8)	40 (41.2)	
Non-Hispanic White/ Other	99 (36.1)	65 (65.7)	34 (34.3)	
Perceived risk of CC M (SD)				<0.001
Same/lower risk	232 (86.90)	147 (63.4)	85 (36.6)	
Higher risk	35 (13.10)	11 (31.4)	24 (68.6)	
Perceived benefits M (SD)	4.01 (1.08)	3.69 (1.12)	4.49 (0.83)	<0.001
HPV knowledge M (SD)	8.03 (4.16)	7.33 (4.25)	9.07 (3.80)	<0.001

Note: Double protected: UTD with CC screening and receipt of HPV vaccine; Unprotected: Not UTD AND not vaccinated. Abbreviations: CC = cervical cancer; HPV = human papillomavirus; UTD = up to date, M = mean, SD = standard deviation.

**Table 3:** Correlations of being double protected: unadjusted and adjusted odds ratios.

Variable	Crude OR (95% CI)	*p*-Value	Adjusted OR (95% CI)	*p*-Value
Age (years)	0.927 (0.891, 0.965)	0.001	0.932 (0.890, 0.976)	0.003
Insured	0.230 (0.103, 0.512)	0.001	0.841 (0.906, 5.932)	0.080
≥1 medical visit in the past year	7.112 (3.235, 15.633)	0.001	1.425 (1.737, 9.946)	0.001
Higher perceived CC risk	3.773 (1.761, 8.084)	0.001	1.295 (1.413, 9.433)	0.008
Perceived benefits of CC screening	2.285 (1.714, 3.045)	0.001	0.675 (1.451, 2.659)	0.000
HPV knowledge	1.112 (1.045, 1.182)	0.001	0.082 (1.007, 1.170)	0.032

Abbreviations: UTD = up to date; CC = cervical cancer; HPV = human papillomavirus; OR = odds ratio; CI = confidence interval.

## Data Availability

Data is available upon reasonable request from Erika Biederman, PhD, RN at emcconne@iu.edu.

## Abbreviations

The following abbreviations are used in this manuscript:

HPV	Human Papillomavirus
CC	Cervical cancer
UTD	Up to date
USPSTF	United States Preventive Services Task Force
HBM	Health Belief Model
Pap	Papanicolaou
